# Nigerian physiotherapists’ knowledge, current practice and perceptions of their role for promoting physical activity: A cross-sectional survey

**DOI:** 10.1371/journal.pone.0266765

**Published:** 2022-05-10

**Authors:** Bashir Bello, Sandra Elaine Hartley, Gillian Yeowell

**Affiliations:** 1 Department of Physiotherapy, Faculty of Allied Health Sciences, Bayero University Kano, Kano, Nigeria; 2 Department of Health Professions, Faculty of Health and Education, Manchester Metropolitan University, Manchester, United Kingdom; Prince Sattam Bin Abdulaziz University, College of Applied Medical Sciences, SAUDI ARABIA

## Abstract

**Background:**

Nigeria has the highest rates of physical inactivity in Africa. As physical inactivity is a leading global risk factor for non-communicable diseases (NCD), physical activity promotion is a strategy for their mitigation. Physiotherapists are already ideally situated to undertake this role and can assist in the reversal of NCD. Gaining insight into how physiotherapists in Nigeria perceive their role in relation to physical activity promotion is needed to ensure this undertaking will be effective. This national survey aimed to investigate Nigerian physiotherapists’ knowledge and current practice for promoting physical activity across Nigeria and perceptions of their role related to this.

**Methods:**

Following ethical approval, a cross-sectional, online questionnaire survey design was employed to investigate the aim. 330 qualified physiotherapists, working across Nigeria were recruited. Internal consistency of the survey was examined using Cronbach’s alpha. Descriptive statistics were used to analyse closed questions. Thematic content analysis was used to analyse open-ended questions. Chi-square inferential statistic was used to investigate the association between variables with alpha interpreted at a level of 0.05.

**Results:**

The internal consistency of the questionnaire survey was good overall (Cronbach Alpha α = 0.71). 330 physiotherapists participated. 99.4% agreed that discussing the benefits of a physically active lifestyle with patients is part of their role. However, over 60% did not feel confident in suggesting specific physical activity programs for their patients. 59.7% were aware of one or more physical activity guideline. However, only 49.1% were incorporating it into their practice. 85.5% felt that developing a physical activity guideline specifically for Nigeria would promote physical activity. 63.3% of respondents did not use any resource in promoting physical activity. An association was found between the physiotherapist’s awareness of physical activity guidelines and male sex (χ^2^ = 8.95, df = 2, p = 0.01).

**Conclusion:**

Whilst most physiotherapists had a positive perception of their role in promoting physical activity, translating this into practice would seem to be challenging. A systems approach to physical activity health promotion is recommended with the need for a commitment by the Nigerian Government to the development and implementation of national guidelines. Incorporating more training in physiotherapy education could foster more confidence in the delivery of these guidelines. Greater use of resources and working with community organisations could help to optimise physical activity uptake in Nigeria.

## Background

Physical inactivity is one of the leading global risk factors for non-communicable diseases (NCD) such as cardiovascular disease, cancer and diabetes as well as death [1, 2]. Conversely, engaging in regular physical activity besides prevention can also be effective in the treatment of NCD [3] and help to improve quality of life and mental wellbeing [4]. Physical inactivity globally is estimated to cost approximately US$ 54 billion per year in direct health care costs with an additional US$ 14 billion attributable to lost productivity [5]. Although disease burden is claimed to be more of a concern for low and middle income countries, with less national revenue per capita, the financial strain can also be considerable [6].

Worldwide, 1 in 4 adults [7], and three quarters of adolescents do not meet the global recommendations for physical activity [8]. In sub-Saharan African, the World Health Organization (WHO) have estimated there are around three million deaths related to physical inactivity [9]. African countries, including Nigeria, bear a disproportionately higher burden of these deaths. This is linked to increased urbanization and economic growth, with a consequent increase in unhealthy lifestyles and sedentary behaviour [10, 11]. Nigeria, with over 200 million citizens has the largest population in Africa [12]. It is estimated that 50 million of its population are not engaging in sufficient weekly physical activity, and as such, Nigeria has the highest rates of physical inactivity on the continent [12]. Furthermore, one quarter of all male and one third of female deaths in Nigeria have been attributed to NCD [13].

Increasing physical activity is a recommended strategy for controlling NCD [3]. As such, as part of the Global Action Plan for the prevention and control of NCD, WHO member states have agreed a 10% reduction in the prevalence of insufficient physical activity by 2025 as one of the nine global targets to improve the prevention and treatment of NCD [14]. However, it has been reported that progress in achieving this has been slow and off target [7, 12], therefore more needs to be done to promote physical activity.

Physiotherapists as experts in movement and exercise, especially in the management of NCDs, are already ideally situated to undertake this role and therefore have a pivotal position in promoting and managing physical activity [15]. As the health professionals with expertise in prescribing exercise for health, physiotherapists can assist in the reversal of NCD and significantly contribute to reducing their global burden [16].

Whilst physiotherapists may be well placed to promote physical activity, gaining more insight into how physiotherapists perceive their role in relation to this is needed to ensure this strategy will be effective. However, the perception of the physiotherapist’s role and practice towards physical activity promotion in Nigeria has not been fully investigated. Studies in this area from Western, high-income countries (for example, Lowe et al. [17]; Freene et al. [18]) may not be applicable to low- and low-middle income African countries such as Nigeria. Moreover, there is limited research on this issue in Nigeria, with the studies undertaken in specific regions and states [19–22], which may not capture the diversity that exists across the country. Currently, no national study has been undertaken that has investigated the perceptions, knowledge, and current practice of the physiotherapist’s role of the physiotherapist in Nigeria in relation to physical activity promotion. By gaining an understanding of Nigerian physiotherapists’ knowledge and current practice nationally, recommendations can be made to enable physiotherapists to enhance their role as promoters of physical activity and assist Nigeria in meeting its global commitments to reducing NCD [23]. Therefore, this national survey aimed to investigate Nigerian physiotherapists’ knowledge and current practice for promoting physical activity across Nigeria and perceptions of their role related to this.

## Methods

### Ethics

Ethical approval was granted from Bayero University’s College of Health Sciences, Kano, Nigeria, ethics committee (reference: BUK/CHS/HREC/132).

### Study design

A cross-sectional, questionnaire survey design was employed to explore physiotherapists’ perceptions, knowledge, and current practice methods for promoting physical activity in Nigeria. STROBE reporting guidelines for observational studies was used to report the study [24].

### Participants

Participants were eligible to take part if they were qualified physiotherapists, working in a private or government-based institution, in any speciality, in Nigeria. Nigeria is made up of 36 states and its Federal Capital Territory, Abuja. The states are grouped into six geopolitical zones (North-east, North-west, North-central, South-west, South-east, South-south), each having different cultural traditions and religious beliefs. To detect a change in the proportion of responses between variables in this study using Chi Squared, an estimated sample of 101 and 137 was required to ensure an alpha of 0.05 with a power of at least 0.80 using G*Power [25]. The estimated sample size was based on an effect size (w = 0.24 and 0.28) derived from questions related to promoting physical activity in a similar study by Aweto [20]. As not all data was statistically analysed, we also considered the degree of confidence in responses using an online tool with 95% confidence intervals, a 5% margin of error and based on a 63.3% population reach [20]; the estimated sample for this aspect of the study was 330.

### Procedure

The survey tool was developed using Google Forms software. The questionnaire was presented in two sections (S1 Appendix). The first section was adapted from a previous questionnaire used to investigate the promotion of physical activity by physical therapists in Australia [26]. It was modified to make it culturally appropriate to Nigeria. This section investigated the following topics: perceptions of the role of physiotherapy in promoting physical activity, knowledge of physical activity guidelines, current practice of physical activity promotion and the perceived barriers and facilitators to this. Most questions were closed with finite choice answers. The second section obtained participants’ socio-demographic information.

The questionnaire survey was pretested by the research team (GY, SH) to ensure the functionality of the online survey. It was then piloted on five specialist physiotherapists involved in health promotion across Nigeria [27]. Their specialities included orthopaedic & sports physiotherapy, neuro-physiotherapy, women’s health physiotherapy, community health & ergonomics physiotherapy, and cardiorespiratory physiotherapy. The physiotherapists included three females and two males with an average age of 38 years and a mean working experience of seven years. The testers agreed that the questionnaire was acceptable and straightforward to use, and they offered suggestions for improvement. They proposed that some parts of the questionnaire be omitted as these were not relevant to physiotherapy in Nigeria and certain questions should be reworded to be more specific to the Nigerian context. Following re-piloting, only minor changes were made in response to feedback about the consistency of wording in some questions. The internal consistency was also checked using a sample of 18 Nigerian physiotherapists who were purposively sampled to reflect a range of genders, work speciality and years of practice experience.

The survey was live between October 2020 to February 2021. The link to the survey was distributed using purposive sampling via the Nigeria Society of Physiotherapy (NSP), WhatsApp groups, and other professional physiotherapy social media networks for each state and region across Nigeria. Snowball sampling was then used whereby respondents were requested to forward the survey link to their physiotherapy colleagues. Reminders were sent every 2 weeks during the period the survey was live to ensure maximum response until the a priori sample size was achieved.

### Data analysis

Before distributing the questionnaire to potential participants, internal consistency was measured using Cronbach’s alpha. Cronbach’s alpha gives scores ranging from 0 to 1, with high alpha values indicating a high degree of interrelatedness among items and values between 0.70–0.95 are considered good [28]. Once assessed, data were collected through Google Forms online survey. The responses were exported to Excel (Microsoft) and transferred to Statistical Packages for the Social Sciences (version 25, IBM SPSS< Armonk, NY) for analysis. Descriptive statistics were used to present the demographic data and to summarize the closed questions, which were presented using percentages and frequency distributions. Chi-square inferential statistic was used to investigate the association between physiotherapists’ socio-demographic variables and promoting physical activity; with alpha interpreted at a level of 0.05. Thematic content analysis was used to analyse open-ended questions, and the results were interpreted in a narrative format.

## Results

The internal consistency of the questionnaire survey ranged from moderate to excellent (Cronbach Alpha α = 0.6 to 1.0) for sub-domains and α = 0.71 for the entire instrument indicating good international consistency overall. All returned questionnaires were analysed. The lead author (BB) was responsible for data processing and management. As the online survey did not allow a respondent to move to the next question until previous sections were completed, all compulsory questions were answered by all respondents. Some questions included an optional open text box to add comments (S1 Appendix), this was not used by all respondents, therefore, total response rates for these optional open text questions vary. Three hundred and thirty (330) physiotherapists participated in this study. The participants’ ages ranged from 20 to 63 years, with a mean age of 35.4 (SD 8.05) years (Table 1). The majority of the respondents (n = 100, 30.3%) were from North-west Nigeria, with a similar percentage (approx. 15%) responding from North-central, South-east and South-west Nigeria. The North-east had the fewest respondents (n = 36, 10.9%). Most respondents were male (58.9%), had an entry-level bachelor’s degree (49.1%), mainly worked in a government hospital setting (73.6%), and had nine or fewer years of practice experience (56.1%), with most (36.5%) working in the orthopaedic and manual therapy specialty.

**Table 1:** Sociodemographic characteristics of participants.

Sociodemographic characteristics n = 330	No. of respondents (%)
**Age (Years)**	
20–29	91(27.6)
30–39	137(41.5)
40–49	79(23.9)
50–59	22(6.7)
≥60	1 (0.3)
**Gender**	
Male	193(58.5)
Female	137(41.5)
**Years of practice**	
< 5	93(28.2)
5–9	92(27.9)
10–15	75(22.7)
16–20	35(10.6)
21–25	19(5.8)
26–30	11(3.3)
>30	5(1.5)
**Highest qualification**	
Bachelor’s	162(49.1)
Masters	131(39.7)
PhD	28(8.5)
DPT	9(2.7)
**Work setting**	
Government hospital	243(73.6)
University	43(13.0)
Private practice	35(10.6)
Sports centers	9(2.8)
**Work Specialty**	
Orthopedic & Manual Therapy	118(35.9)
Cardiopulmonary	26(7.6)
Neurophysiotherapy	50(15.2)
Paediatric physiotherapy	31(9.5)
Sports physiotherapy	9(2.7)
Geriatric physiotherapy	7(2.1)
Women’s health	8(2.4)
General physiotherapy	81(24.6)
**Work region**	
North-east	36(10.9)
North-west	100(30.3)
North-central	51(15.5)
South-west	49(14.8)
South-east	51(15.5)
South-south	43(13.0)

Almost all the respondents (99.4%) agreed that discussing the benefits of a physically active lifestyle with patients is a part of a physiotherapist’s role and that they should be physically active to serve as role models for their patients (Table 2). However, over 60% of respondents did not feel confident in suggesting specific physical activity programs for their patients.

**Table 2:** Perception and confidence statements of respondents.

		No. (%) of respondents				
Statement	n	Strongly agree	Agree	Undecided	Disagree	Strongly disagree
Discussing the benefits of a physically active lifestyle with patients is a part of a physiotherapist’s role	330	269 (81.5%)	59 (17.9%)	-	1 (0.3%)	1 (0.3%)
Discussing how patients can increase their physical activity levels is a part of a physiotherapist’s role	330	252 (76.4%)	76 (23%)	2 (0.6%)	-	-
Physiotherapists should promote physical activity in every contact they have with their patient	330	169 (51.2%)	144 (43.6%)	8 (2.4%)	9 (2.7%)	-
Physiotherapists should be physically active to act as a role model for their patients	330	225 (68.2%)	95 (28.8%)	7 (2.1%)	3 (0.9%)	-
I feel confident in giving general advice to patients on living a physically active lifestyle	330	221 (67%)	101 (30.6%)	8 (2.4%)	-	-
I feel confident in suggesting specific physical activity programs for my patients	330	106 (32.1%)	7 (2.1%)	16 (4.8%)	46 (13.9%)	155 (47%)

Sixty percent of respondents said that they were aware of one or more physical activity guidelines (Table 3). However, only 49.1% of the respondents were incorporating it into their practice. The most common physical activity guidelines known and used by the respondents were the WHO physical activity guidelines and the American College of Sports Medicine Guidelines (ACSM).

**Table 3:** Knowledge and current practice statements of respondents.

		No. (%) of respondents		
Statement	n	YES	NO	MAYBE
Are you aware of any of the physical activity guidelines?	330	197 (59.7%)	70 (21.2%)	63 (19.1%)
Do you use any of these guidelines?	330	162 (49.1%)	103 (31.2%)	65 (19.7%)
I recommend physical activity if a patient’s health condition demands it	330	254 (77%)	71 (21.5)	5 (1.5%)
I recommend physical activity even to a healthy person to keep an active lifestyle	330	254 (77%)	5 (1.5%)	71 (21.5%)
I initiate conversations about physical activity with all my patients	330	207 (62.7%)	7 (2.1%)	116 (35.2%)
I assess my patient’s physical activity status irrespective of their health needs	330	120 (36.4%)	16 (4.8%)	194 (58.8%)
As part of your undergraduate physiotherapy course did you learn about promoting physical activity for health	330	232 (70.3%)	84 (25.5%)	14 (4.2%)

**Fig 1:**
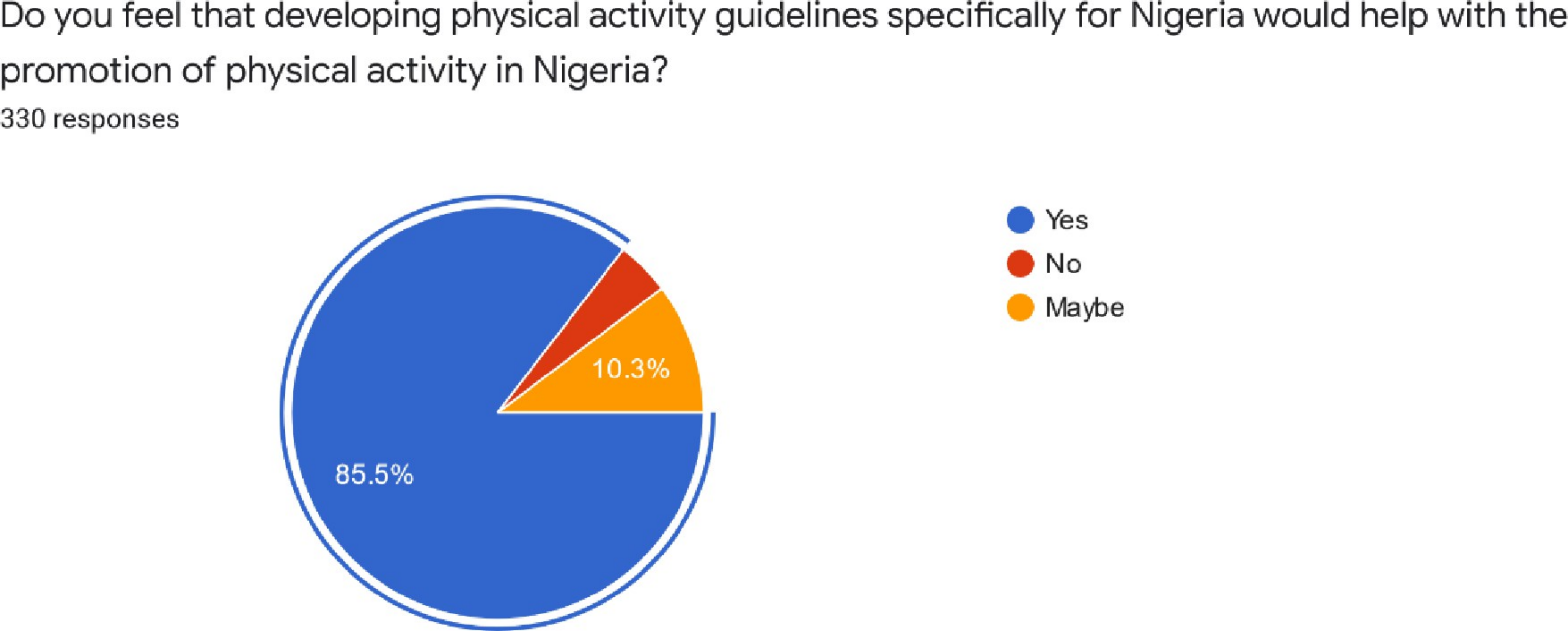
Responses on the need to develop physical activity guidelines specifically for Nigeria.

The majority of the respondents (85.5%) felt that developing a physical activity guideline specifically for Nigeria would promote physical activity in Nigeria (Fig 1). Analysis of the open text comments revealed that participants’ most typical reasons for this were that it would be: more culturally specific ‘*Developing guidelines that fit our environment*, *culture and socioeconomic status will improve engagement’;* lead to better client outcomes *‘It might increase the level of health status of many Nigerians and hence help in reducing hospitalization thereby reducing the cost of living’*; promote compliance and removal of barriers *‘This will help in removing barriers such as language*, *culture as well as religion’*.

**Fig 2:**
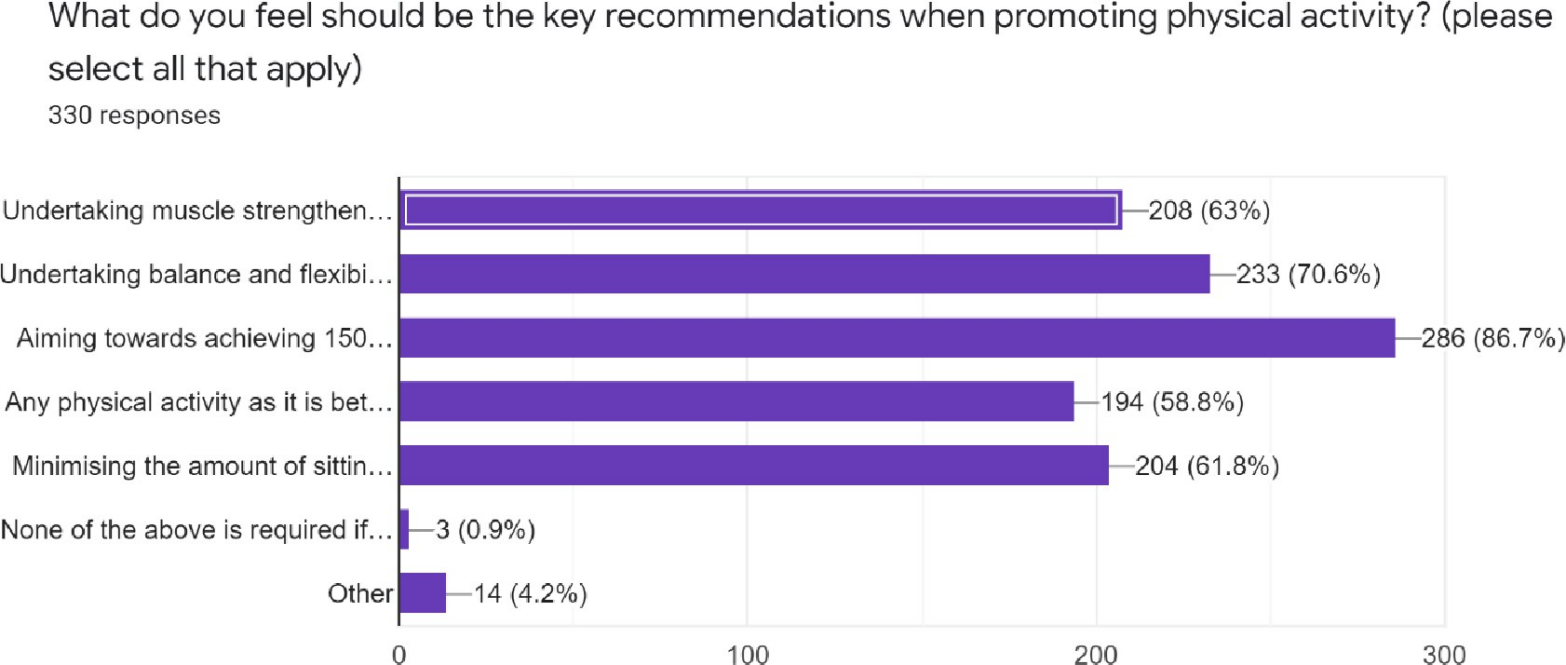
Key recommendations when promoting physical activity.

Most respondents (58.8–86.7%) felt that the key recommendations when promoting physical activity were: undertaking muscle strengthening, balance and flexibility exercises, achieving 150 mins/week of physical activity, and minimizing long sitting periods per day (Fig 2).

**Fig 3:**
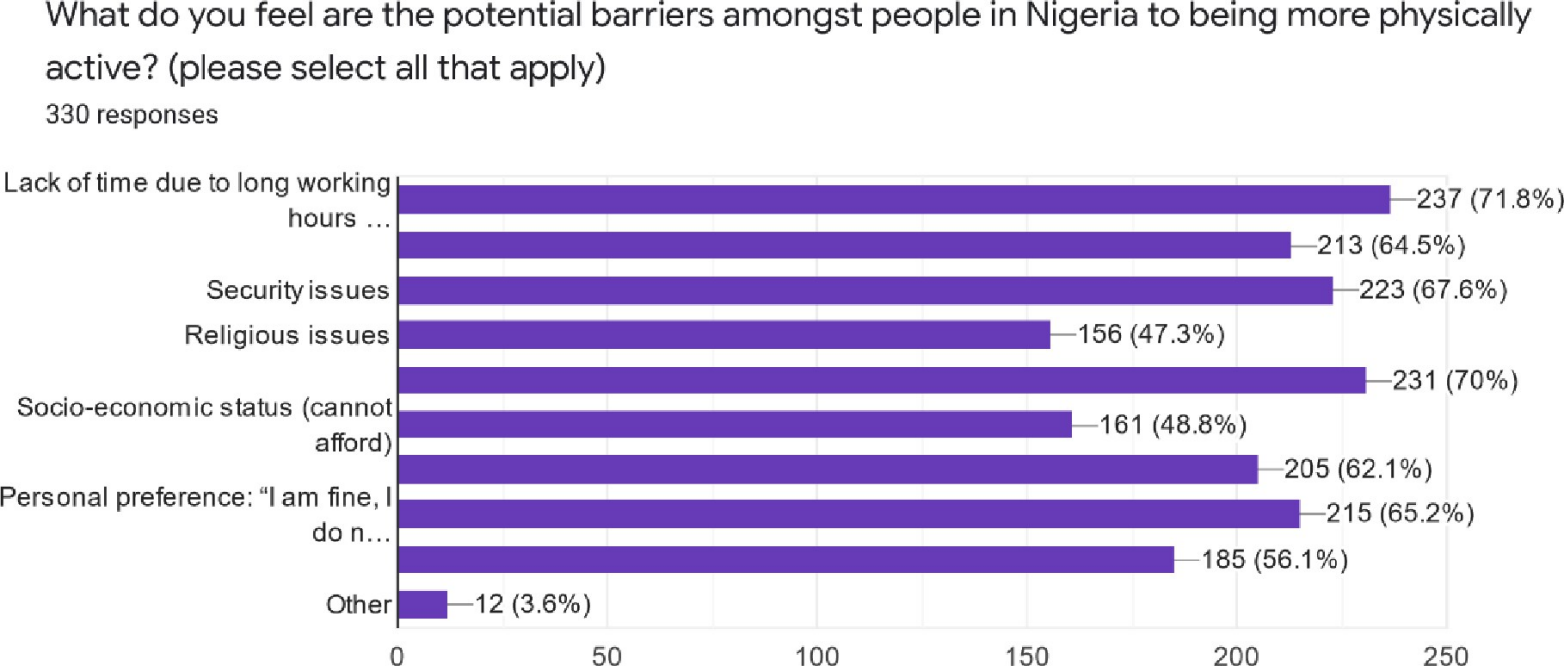
Barriers to physical activity promotion in Nigeria.

The most common potential barriers stated by respondents for people not being physically active included lack of time, security, religious and economic issues, perception of ‘*being fine or healthy*’ and thus did not need it (Fig 3).

Analysis of the open text comments regarding the barriers to being more physical activity revealed n = 270 of the respondents felt that education of the general population was needed to increase their knowledge of the benefits of physical activity *‘Creating more awareness by health practitioners to make people understand that it’s the cheapest medicine in managing and preventing diseases’*. Additional factors mentioned were: enhancing security and environmental factors, *‘Improved security for the average Nigerian will help a lot’;* Accessibility and affordability, ‘*Walkways and sporting centres should be made more accessible and affordable’*; cultural factors ‘*Engaging opinion leaders such as religious and traditional leaders on the need to encourage their followers on the benefits of engaging in physical activity’*, and lifestyle modifications ‘*Incorporate it in workplace’*.

**Fig 4:**
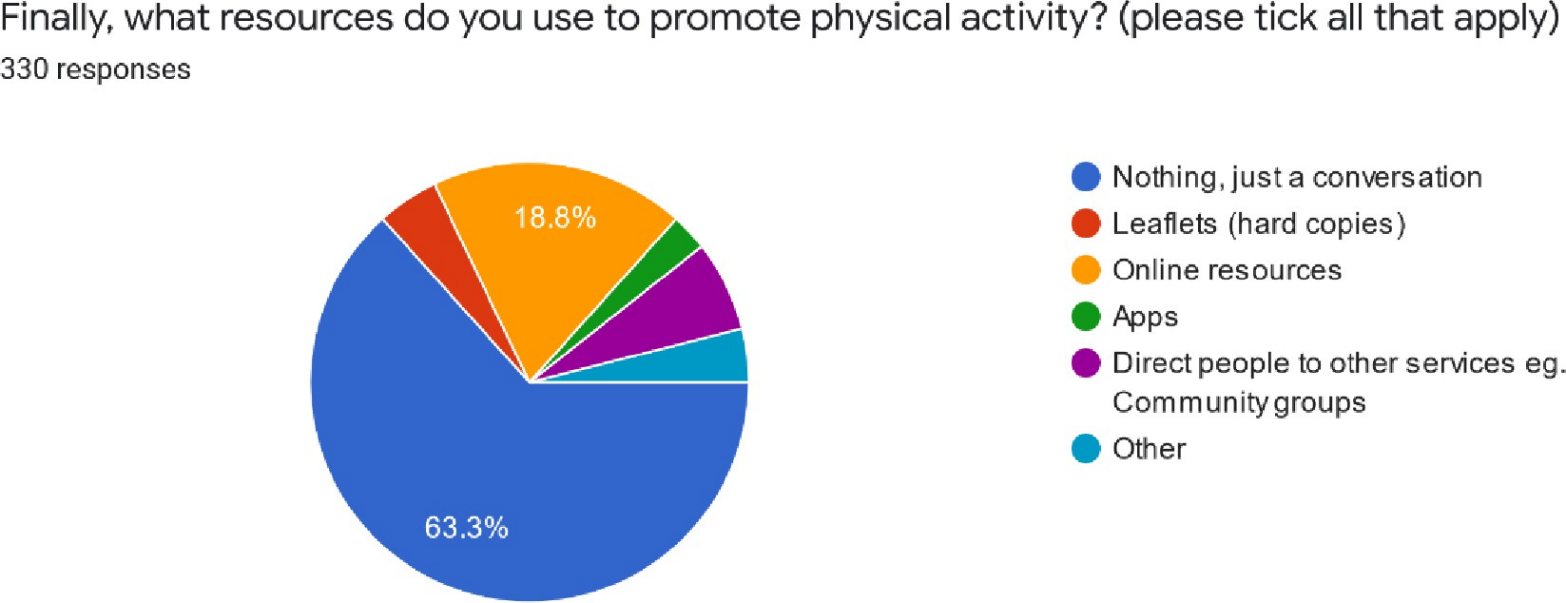
Pie chart of various resources used to promote physical activity.

Surprisingly, 63.3% of respondents do not use any resource in promoting physical activity, rather used conversation alone (Fig 4). ‘Other’ resources (3.9%) that respondents highlighted were verbal explanations and practical demonstrations.

An association was found between the physiotherapist’s awareness of physical activity guidelines and male sex (χ^2^ = 8.95, df = 2, p = 0.01). However, no associations were found between promoting physical activity and region; learning about physical activity promotion as an undergraduate; and area of work (all p > 0.05).

## Discussion

Physiotherapists in our study had a positive disposition towards physical activity promotion with almost all agreeing that discussing the benefits of a physically active lifestyle with patients is a part of a physiotherapist’s role. However, approximately 40% claimed they were not aware or were uncertain about specific physical activity guidelines, and fewer than 50% used any physical activity guidelines in their current practice. This reflects previous research findings in Northern Nigeria [22] and other countries [17, 29, 30], which have demonstrated physiotherapists’ willingness to promote physical activity, even though many lacked knowledge of physical activity guidelines. For those who were familiar with the guidelines, the WHO and the ACSM guidelines were the most commonly known and used. The WHO guidelines have been developed for use in high, middle and low-income countries and are promoted globally to reduce sedentary behaviour in various populations [23]. It is likely that this is why these were one of the most well-known among the physiotherapists in our study. Although including information appropriate to low and lower-middle income countries such as Nigeria improves the WHO guidelines relevancy, tailoring these specifically to their population’s needs would enhance their applicability further.

Indeed, appropriateness of guidelines was clearly a concern for physiotherapists in our study as the majority of respondents (85.5%) acknowledged that developing physical activity guidelines specifically for Nigeria would help promote physical activity. Highlighting the need for Nigerian physical activity guidelines is consistent with previous research to enhance physical activity promotion in this country [20]. Respondents in our study, suggested that developing more culturally specific guidelines tailored to Nigeria could help address barriers such as those due to language, culture and religion, thereby promoting compliance and thus leading to better client outcomes in this population. In fact, the 2015 Nigerian National Strategic Plan of Action on NCD proposed the development and implementation of national guidelines on physical activity for health [31], however; these have not yet come into fruition. As a Member State of the United Nations (UN), Nigeria has adopted the 2030 Agenda for Sustainability Development [32]. Core to this, are the 17 Sustainable Development Goals (SDG), with Goal 3 being ‘to ensure healthy lives and promote well-being for all at all ages’ [32]. A review in 2020 on the progress Nigeria has made towards these goals has emphasised that more government investment in public health and delivery of national initiatives is required [33]. As such, more commitment from the government to address determinants of health such as physical inactivity is urgently needed, as it will help it meet its SDG targets [32].

In our study, whilst most felt confident in providing general advice on living a physically active lifestyle, the majority of physiotherapists did not feel confident in suggesting specific physical activity programmes for their clients. This resonates with earlier findings particular to Nigeria [20]. Undergraduate and postgraduate curricula in relation to health promotion in Nigeria has previously been highlighted as inadequate [34]. Therefore, the lack of confidence in implementing specific physical activity programmes into routine clinical practice may be related to lack of training and continuing education of physiotherapists in physical activity promotion. Incorporating more physical activity training within the physiotherapy profession in Nigeria could consequently, foster more self-assurance in the delivery of specific physical activity programs and help to optimise physical activity promotion for health improvement [35]. However, to amplify the public health message and help to facilitate a change in practice, national backing is also required [36]. For example, in the United Kingdom (UK), ‘Making Every Contact Count’ and ‘Moving Medicine’ are both government-endorsed initiatives that guide, support and champion health professions in promoting health behaviour change, such as being physically active, to improve people’s health and wellbeing [35, 37]. Both enterprises provide toolkits and other evidence-based resources to facilitate transference into practice by health professionals during routine conversations with their clients [37, 38]. Educational resources are also provided to foster the embedment of health promotion into health professionals’ undergraduate and postgraduate training [35]. Creating national campaigns in Nigeria such as these, and developing effective communication channels to promote awareness of, and access to, resources by physiotherapists could help to address the lack of confidence reported by some in our study.

The most common barriers to physical activity highlighted by the physiotherapists in our study included time, security, religion and economic factors. Wider influences such as socio-economic, cultural and environmental conditions are known determinants of physical activity and sedentary behaviours [39, 40]. These are particularly important factors in a country such as Nigeria where 40% of the population, which equates to over 82.9 million Nigerians, are deemed to be living in poverty [41]. Furthermore, there are large inequalities in education attainment and healthcare access [42, 43], and substantial regional variation, with higher rates of poverty in the North-west region of Nigeria [44]. Interestingly, no association was found between promoting physical activity and region in our study. This may suggest that factors to promoting physical activity promotion are more complex and nuanced. Previous studies have shown that effective physical activity promotion requires implementing culturally sensitive, multi-component interventions that make it easier for people to stay active [45, 46]. Recognising the diverse influences on physical activity promotion, through a systems approach, whereby both national and community campaigns across multi-sectors are utilized, would therefore seem essential [12, 47]. This is also more likely to foster collective advocacy for policy implementation as well as develop local tailored interventions that support community socio-economic, environmental and cultural needs [47, 48]. For example, the African Physical Activity Network, which includes stakeholders from private, public and the third sector [49], are ideally placed to drive physical activity initiatives across the country. However, more support and investment from national government would help to optimise the opportunities they could offer.

In our study, as elsewhere [29], most physiotherapists did not use any resources in promoting physical activity and preferred to do this through conversation alone. Physical activity promotion through patient education could provide a cost-effective intervention, particularly for managing people with chronic conditions [50]. However, translating physical activity promotional messages into practice have been found to be challenging [51, 52]. Therefore, incorporating other means to aid conversations could optimise its effectiveness. Of the physiotherapists in our study, 18.8% used online resources and only 4.5% used leaflets. Including visual aids, such as infographics of the physical activity guidelines, could help to facilitate physical activity promotion to patients, as well as support health professionals in providing appropriate information [52].

Evidence has also highlighted the benefits of technology for enhancing physical activity promotion including smartphone apps and activity trackers [53, 54]. However, in our study, apps were the least favoured resource used. The utilisation of technology could lead to widening inequities for those who lack digital literacy or the financial means to access these resources [55]. Nevertheless, internet and smartphone use amongst the Nigerian population is continuing to grow [56]. Therefore, more use of technology and apps could offer further opportunities to support physical activity promotion. Moreover, providing apps that are free and can be tailored to individual needs should be considered to improve inclusivity [57].

In our study only 6.7% of physiotherapists directed people to other services, such as community groups. Working with community organisations that are embedded locally and hence offer more insight into their population’s needs, could foster more effective ways to optimise physical activity promotional campaigns [58]. Previous research has also highlighted the potential of community health workers undertaking public health promotion in Africa [59]. Physiotherapists could also collaborate with health workers to endorse physical activity within their locality. Furthermore, it has be found that facilitating communities to take ownership of the health initiatives delivered in their area improves safe access to services and helps to build trust with health care organisations [23].

It is reassuring that our study found that most physiotherapists perceived they should be physically active to serve as role models for their patients. Previous research has found that patients may be more motivated to change their health behaviour if the health professional is seen as a role model in that behaviour [60, 61]. Therefore, it is important that physiotherapists are aware of the impact that role modelling has on client’s uptake of physical activity. Designing workplace initiatives that foster healthcare workers engagement in healthy behaviours, including physical activity, would not only facilitate physiotherapists’ ability to become role models but also enhance their health and hence the population health of Nigeria [62].

### Strengths and limitations

This is the first national survey to investigate Nigerian physiotherapists’ knowledge and current practice for promoting physical activity across Nigeria and perceptions of their role related to this. The survey was found to have good international consistency overall. However, this study has some limitations. The majority of respondents (30%) were from the North-west zone. This may be due to the primary researcher working and residing in the location, which may have increased colleagues from that region to participate in the study. This may indicate selection bias in the geopolitical zones with the highest number of participants. In addition, social desirability bias could lead respondents to answer the questions as perceived appropriate. However, as the survey was anonymous this may have helped mitigate this. The online format could also allow physiotherapists to search online before responding to certain questions such as for the questions around knowledge of physical activity guidelines. However, the lack of knowledge around physical activity guidelines may suggest this was not the case. Finally, whilst male sex was found to be significantly associated with physiotherapist’s knowledge of physical activity guidelines, this may be due to the majority of respondents surveyed being male, rather than males having a greater knowledge of the guidelines.

## Conclusion

This study found that physiotherapists in Nigeria had a positive perception of their role in promoting physical activity among people in Nigeria. However, translating physical activity promotion into practice would seem to be challenging. Incorporating more training in physiotherapy education could foster more confidence in the delivery of these guidelines. The use of technology, visual aids, including infographics of guidelines and working with community organisations could help tailor promotional campaigns to the population’s needs to optimise physical activity uptake in Nigeria. Finally, a systems approach to physical activity health promotion that includes both national and community campaigns across multi-sectors is recommended.

## Supporting information

S1 Appendix Questionnaire survey. (DOCX)
