# Supplementary material for: Nigerian physiotherapists’ knowledge, current practice and perceptions of their role for promoting physical activity: A cross-sectional survey
Source: PLoS One. 2022 May 10;17(5):e0266765. doi: 10.1371/journal.pone.0266765 (PMC9089902; doi:10.1371/journal.pone.0266765)
Supplement: S1 Appendix — (DOCX) [file pone.0266765.s001.docx]

PHYSIOTHERAPISTS' PHYSICAL ACTIVITY QUESTIONNAIRE

* Required

PART 1

To what extent do you agree or disagree with the following statements:

1.Discussing the benefits of a physically active lifestyle with patients is a part of a physiotherapist’s role *

- Strongly Agree
- Agree
- Undecided
- Disagree
- Strongly Disagree

2. Discussing how patients can increase their physical activity levels is a part of a physiotherapist’s role *

- Strongly Agree
- Agree
- Undecided
- Disagree
- Strongly Disagree

3. Physiotherapists should promote physical activity in every contact they have with their patient *

- Strongly Agree
- Agree
- Undecided
- Disagree
- Strongly Disagree

4. Physiotherapists should be physically active to act as a role model for their patients *

- Strongly Agree
- Agree
- Undecided
- Disagree
- Strongly Disagree

5. I feel confident in giving general advice to patients on living a physically active lifestyle *

- Strongly Agree
- Agree
- Undecided
- Disagree
- Strongly Disagree

6. I feel confident in suggesting specific physical activity programs for my patients *

- Strongly disagree
- Disagree
- Neutral
- Agree
- Strongly agree

7. Are you aware of any of the physical activity guidelines? *

- Yes
- No
- Maybe

If yes, which guidelines are you aware of?

Your answer

8. Do you use any of these guidelines? *

- Yes
- No
- Maybe

If yes, which guidelines do you use?

Your answer

9. Do you feel that developing physical activity guidelines specifically for Nigeria would help with the promotion of physical activity in Nigeria? *

Yes

No

Maybe

If yes / no, why?

Your answer

10. What do you feel should be the key recommendations when promoting physical activity? (please select all that apply) *

- Undertaking muscle strengthening on at least 2 days per week
- Undertaking balance and flexibility exercises on at least 2 days per week
- Aiming towards achieving 150mins/week of physical activity that increases the person’s heart rate
- Any physical activity as it is better than none
- Minimising the amount of sitting for long periods
- None of the above is required if a person is healthy
- Other

If you have selected Other, please specify:

Your answer

11. I recommend physical activity if a patient’s health condition demands it *

- Always
- Sometimes
- Never

12. I recommend physical activity even to a healthy person to keep an active lifestyle *

- always
- Sometimes
- Never

13. I initiate conversations about physical activity with all my patients *

- Always
- Sometimes
- Never

14. I assess my patient’s physical activity status irrespective of their health needs *

- Always
- Sometimes
- Never

15. What do you feel are the potential barriers amongst people in Nigeria to being more physically active? (please select all that apply) *

- Lack of time due to long working hours or additional responsibilities
- Traditional /cultural beliefs
- Security issues
- Religious issues
- Lack of motivation and support from family and friends
- Socio-economic status (cannot afford)
- Environmental reasons: required facilities are too far or ones that are available are not suitable/inadequate
- Personal preference: “I am fine, I do not need to exercise”
- People in Nigeria are not aware of the importance of physical activity for a healthier lifestyle
- Other

If you have selected Other, please specify:

Your answer

16. What do you think can be done to overcome these potential barriers to help promote physical activity? *

Part II

Now a bit about you

17. Are you: *

- Female
- Male
- Prefer not to say
- Other:

18. What is your age in years *

Your answer

19. What is your highest physiotherapy qualification *

- Bachelors’ Graduate
- Masters’ Graduate
- PhD
- DPT
- Other:

20. What year did you qualify as a physiotherapist *

Your answer

21. How many years work experience as a physiotherapist do you have (years/months): *

Your answer

22. As part of your undergraduate physiotherapy course did you learn about promoting physical activity for health (please tick one): *

- Yes
- No
- Maybe

23. In what region of Nigeria do you currently work *

- North East
- North Central
- North West
- South West
- South East
- South South

24. In which setting is your physiotherapy work experience (please tick all that apply) *

- Private clinic
- Government Hospital
- University
- Home visit
- Non - Governmental Organization (NGO)
- Other:

If you have selected Other, please specify:

Your answer

25. What is the main area of physiotherapy you currently work in (please tick one) *

- Orthopaedics / manual therapy
- Paediatrics
- Geriatrics
- Women’s health
- Cardio-pulmonary
- Sports
- Other

If you have selected Other, please specify:

Your answer

26. Finally, what resources do you use to promote physical activity? (please tick all that apply) *

- Nothing, just a conversation
- Leaflets (hard copies)
- Online resources
- Apps
- Direct people to other services eg. Community groups
- Other

If you have selected Other, please specify:

Your answer

THANK YOU FOR PARTICIPATING IN THIS SURVEY

END OF THE QUESTIONNAIRE
